# Hypertensive Effect of Downregulation of the Opioid System in Mouse Model of Different Activity of the Endogenous Opioid System

**DOI:** 10.3390/ijms22084179

**Published:** 2021-04-17

**Authors:** Dominik S. Skiba, Piotr Szczepaniak, Mateusz Siedliński, Piotr Poznański, Marzena Łazarczyk, Kinga Jaskuła, Piotr Religa, Mariusz Sacharczuk, Zbigniew Gaciong

**Affiliations:** 1 Department of Experimental Genomics, Institute of Genetics and Animal Biotechnology, Polish Academy of Sciences, Postępu 36A, Jastrzębiec, 05-552 Magdalenka, Poland; d.skiba@igbzpan.pl (D.S.S.); p.poznanski@igbzpan.pl (P.P.); m.lazarczyk@igbzpan.pl (M.Ł.); k.jaskula@igbzpan.pl (K.J.); p.religa@igbzpan.pl (P.R.); m.sacharczuk@igbzpan.pl (M.S.); 2 British Heart Foundation Centre for Excellence, Institute of Cardiovascular and Medical Sciences, University of Glasgow, University Place 126, Glasgow G12 8TA, UK; 3 Department of Internal and Agricultural Medicine, Translational Medicine Laboratory, Collegium Medicum Jagiellonian University, Skarbowa 1, 31-121 Krakow, Poland; piotr.szczepaniak@uj.edu.pl (P.S.); mateusz.siedlinski@uj.edu.pl (M.S.); 4 Faculty of Pharmacy with the Laboratory Medicine Division, Department of Pharmacodynamics, Medical University of Warsaw, Centre for Preclinical Research and Technology, Banacha 1B, 02-091 Warsaw, Poland; 5 Department of Internal Medicine, Hypertension and Vascular Diseases, Medical University of Warsaw, Banacha 1A, 02-091 Warsaw, Poland

**Keywords:** opioid system, vascular function, guanylyl cyclase, blood pressure

## Abstract

The opioid system is well-known for its role in modulating nociception and addiction development. However, there are premises that the endogenous opioid system may also affect blood pressure. The main goal of the present study was to determine the impact of different endogenous opioid system activity and its pharmacological blockade on blood pressure. Moreover, we examined the vascular function in hyper- and hypoactive states of the opioid system and its pharmacological modification. In our study, we used two mouse lines which are divergently bred for high (HA) and low (LA) swim stress-induced analgesia. The obtained results indicated that individuals with low endogenous opioid system activity have higher basal blood pressure compared to those with a hyperactive opioid system. Additionally, naloxone administration only resulted in the elevation of blood pressure in HA mice. We also showed that the hypoactive opioid system contributes to impaired vascular relaxation independent of endothelium, which corresponded with decreased guanylyl cyclase levels in the aorta. Together, these data suggest that higher basal blood pressure in LA mice is a result of disturbed mechanisms in vascular relaxation in smooth muscle cells. We believe that a novel mechanism which involves endogenous opioid system activity in the regulation of blood pressure will be a promising target for further studies in hypertension development.

## 1. Introduction

The endogenous opioid system has long been almost exclusively concerned with its effects on analgesic and antinociceptive phenomena [1]. However, it was found relatively early that exogenous opioids may exert potent cardiorespiratory effects [2]. Since then, the role of opioid peptides in the autonomic regulation of blood pressure, heart function, and respiration became even more complex [3]. Opioids may be cardioprotective by attenuating ischemic reperfusion injury and decreasing apoptosis in myocytes, causing smaller infarct and ameliorating ventricular function [4]. Opioids naturally exist in opium—a dried latex obtained from the seed capsules of the opium poppy *Papaver somniferum*. Opium may temporarily reduce blood pressure (BP), but it increases the concentration of blood glucose and most blood lipids. Moreover, its long-term use has negative impacts and thus it aggravates diabetes, dyslipidaemia and hypertension [5]. Human studies showed that opium addiction may be significant risk factor for coronary artery disease (CAD) [6]. Interestingly, in human subjects it was found that endogenous opioid activity may contribute to generally reduced pain sensitivity, and perhaps mood reactivity, in those with higher BP [7]. It may be an important link between the altered neuropeptide regulation of pain and altered BP control mechanisms in the early stages of hypertension [8]. On the other hand, studies on spontaneously hypertensive rats (SHRs) showed decreased opioid levels of dynorphin and leu-enkephalin in the brain [9], but not in deoxycorticosterone acetate- and salt-induced hypertension models [10]. The antihypertensive drug, clonidine, which stimulates α2 adrenergic receptors in the brain stem, was reported to increase β-endorphin release from the brains of SHR rats, whereas opioid antagonists blunt the hypotensive effect of clonidine [11]. Opioid peptides and their G protein-coupled receptors (GPCRs) are important regulators within the cardiovascular system, implicated in the modulation of electrophysiological function, heart rate, myocardial inotropy and vascular function. It was found that morphine caused naloxone-reversible relaxant responses in preconstructed rat aortic rings. This effect was, however, not related to nitric oxide release [12]. On the other hand, high doses of morphine impair vascular endothelial function by the increased production of superoxide anions [13]. Another opioid agonist—biphalin, a synthetic non-addictive enkephalin analogue—decreases BP in SHRs [14]. However, another study shows that opioids’ effect on BP level depends on the hypertension model used in the study [15]. Therefore, we aimed to characterize vascular function and BP level in a unique model of mice selected for high and low activity of the endogenous opioid system [16,17]. Mice selected for high (HA) and low (LA) swim stress-induced analgesia (SSIA) are characterized by unique, inherited differences in opioid system activity [18]. HA mice exhibited increased opioid system activity as compared to their LA counterparts and showed enhanced G-protein activity in central nervous system structures involved in pain modulation [16].

During the past four decades, persistently raised BP has been reported in central and eastern Europe, which indicates a maintenance of unfavorable trends towards the risk of hypertension. Among hypertensive patients, those resistant to available drugs account for almost 20% of all cases [19], so that seeking new mechanisms and molecular targets is still a necessary challenge to undertake. In this study, we try to face the unsolved problem emphasizing endogenous opioid system involvement in hypertension development.

## 2. Results

### 2.1. HA and LA Mice Have Respectively High and Low Analgesia Measured by Tail-Flick Test and Hot Plate Test

**Figure 1:**
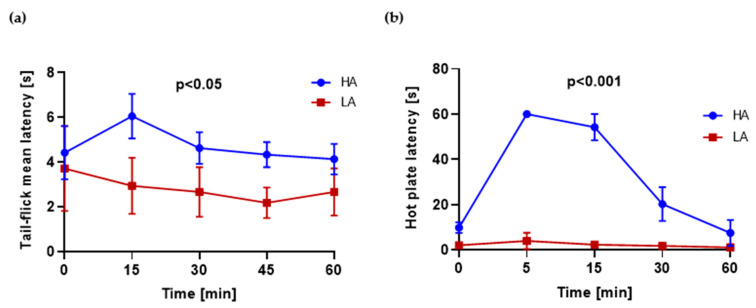
(**a**) Tail-flick latency in HA and LA mice (*n* = 5) in time after swimming, average of three repetitions; (**b**) hot plate test in HA and LA mice, in time after swimming (*n* = 5).

Hot plate (HP) and tail-flick (TF) tests were used to assess analgesia in HA and LA mice. HA mice had significantly higher latency measured 15, 30, 45 and 60 min after swimming measured by the TF test (Figure 1a). The latency of HA mice measured by the HP test after swimming was elevated, reaching the maximum value after 5 min in comparison to LA mice. LA mice were characterized with a constant latency time throughout the whole period (Figure 1b).

### 2.2. Low Activity of Opioid System Manifests Elevated Blood Pressure, without Effect on Endothelium Dependent Vascular Relaxation and Constraction in Aorta

**Figure 2:**
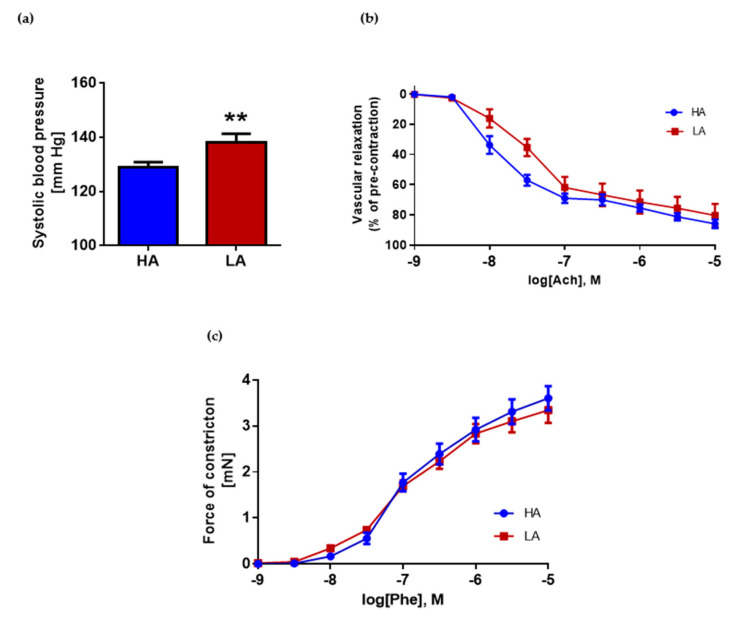
(**a**) Systolic blood pressure in HA and LA mice in basal conditions HA and LA (*n* = 25–27); (**b**) vascular relaxation of aortas from HA and LA mice in response to acetylcholine (*n* = 9–10); (**c**) Phenylephrine-induced vascular constriction in aortas from HA and LA mice (*n* = 9–10). ** *p* < 0.01.

LA mice with decreased activity of the opioid system had significantly higher systolic BPs compared to HA mice with high activity of the opioid system (LA 138.23 ± 3.05 mmHg vs. HA 128.77 ± 2.06 mmHg) (Figure 2a). However, vascular relaxation of the aorta measured by the response to rising concentrations of acetylcholine showed no differences in HA mice compared to LA (Figure 2b). Despite this, statistical analysis of individual concentrations showed differences between groups at logarithmic concentrations of −8 M and −7.5 M. At those concentrations of acetylcholine, the aorta of HA mice relaxed better. Another factor which usually is affected in arteries from hypertensive subjects is vascular constriction. We have not observed differences in constriction force in response to phenylephrine between the LA and HA group (E_max_ LA: 3.35 ± 0.28 mN, E_max_ HA: 3.60 ± 0.26 mN) (Figure 2c). The data were presented as a force of constriction.

### 2.3. Low Activity of Opioid System Causes Worse Endothelium-Independent Relaxation Which Correspond with Decreased Level of Guanylyl Cyclase (Gucy1A3) Despite of Elevated eNOS Expression

**Figure 3:**
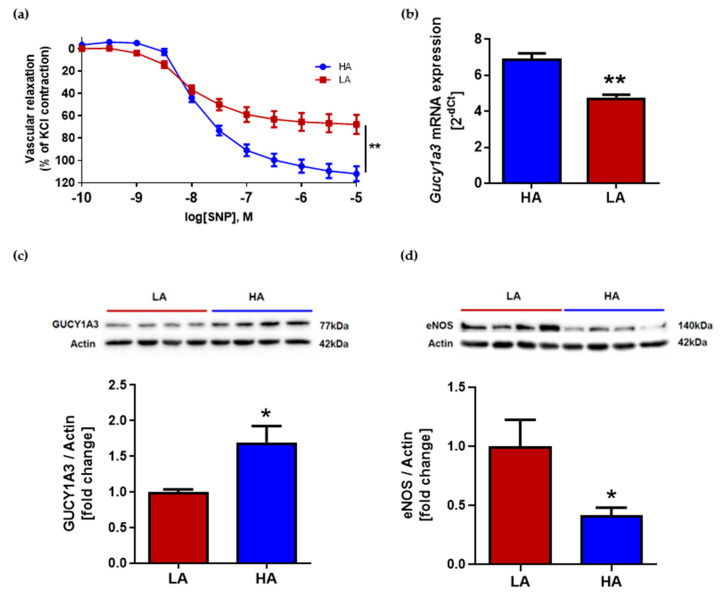
(**a**) Vascular relaxation of aortas from HA and LA mice in response to SNP; (**b**) expression of guanylyl cyclase mRNA in aorta of HA and LA mice (*n* = 8); (**c**) GUCY1A3 protein level in aorta of HA and LA mice (*n* = 4) corrected to actin; (**d**) eNOS protein level in aorta of HA and LA mice (*n* = 4) corrected to actin.* *p* < 0.05, ** *p* < 0.01.

Vascular response to nitric oxide (NO)-donor after NOS (nitric oxide synthase) inhibition by (ω)-nitro-L-arginine methyl ester (L-NAME) was evaluated. Analysis of endothelium-independent relaxation showed differences between groups. The aorta from HA mice had significantly better relaxation in response to sodium nitroprusside (SNP) when compared to LA (Figure 3a). One of the key components involved in endothelium-independent vascular relaxation is guanylyl cyclase. The reduced expression of guanylate cyclase leads to the reduced production of cGMP, and thus impairs endothelial-independent diastolic capacity in the arteries. In our study, the expression of mRNA for Gucy1a3 in aortas was higher in HA mice (HA: 6.86 ± 0.37 vs. LA: 4.67 ± 0.25) (Figure 3b) and corresponded with the lower systolic BP of HA mice when compared to LA. Guanylyl cyclase expression on the protein level was also elevated in HA mice when compared to LA (1.70 ± 0.23 vs. 1.00 ± 0.04) (Figure 3c). Main source of NO for vascular smooth muscle cells is endothelial synthase. Interestingly, endothelium NOS expression on the protein level was decreased in aortas from HA mice in comparison to LA mice (0.41 ± 0.06 vs. 1.00 ± 0.22) (Figure 3d).

### 2.4. Naloxone Treatment Elevates Blood Pressure in Mice with High Activity of Opioid System and Decreases Guanylyl Cyclase mRNA Expression in Aorta

**Figure 4:**
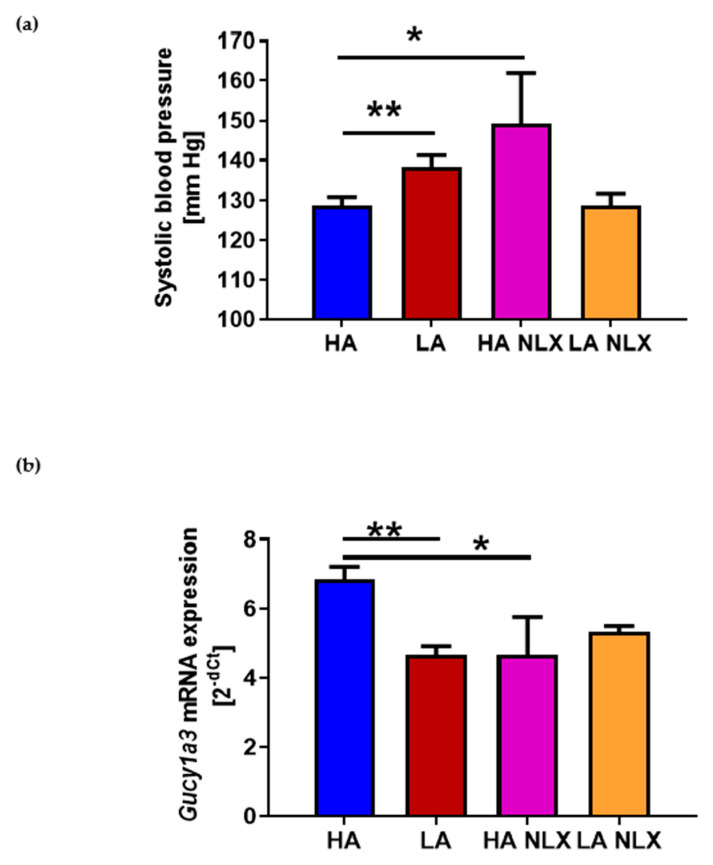
(**a**) Systolic blood pressure in HA and LA mice in basal conditions (*n* = 25–27) and after naloxone treatment (*n* = 4); (**b**) expression of guanylyl cyclase mRNA in aortas of HA and LA mice (*n* = 8) and HA and LA mice treated with naloxone (*n* = 4); * *p* < 0.05, ** *p* < 0.01.

To testify the hypothesis that an observed effect on BP is related to opioid receptors, we performed an experiment where HA and LA mice were treated with non-specific opioid receptor antagonist—naloxone (NLX). The pharmacological blockade of opioid receptors led to the increase in systolic BP in HA mice (HA 128.77 ± 2.06 mmHg vs. HA NLX 149.26 ± 12.62 mmHg) (Figure 4a). Interestingly, we did not observed differences in BP level in naloxone-treated LA mice (LA 138.23 ± 3.05 mmHg vs. LA NLX 128.81 ± 2.88 mmHg) (Figure 4a). Hence, we performed an analysis of guanylyl cyclase of mRNA in those mice, being aware that the expression of that gene was decreased in LA mice. Naloxone treatment in HA mice significantly decreased guanylyl cyclase expression in the aorta (HA: 6.86 ± 0.37 vs. HA NLX: 4.67 ± 1.10) (Figure 4b). Interestingly, NLX treatment had no effect on guanylyl cyclase expression in LA mice (LA: 4.67 ± 0.25 vs. LA NLX: 5.35 ± 0.16) (Figure 4b).

### 2.5. Aortic Segments Treated In Vitro with Naloxone Exert No Changes in Both Endothelium Dependent and Independent Vascular Function in LA and HA Models of Opioid System Activity

**Figure 5:**
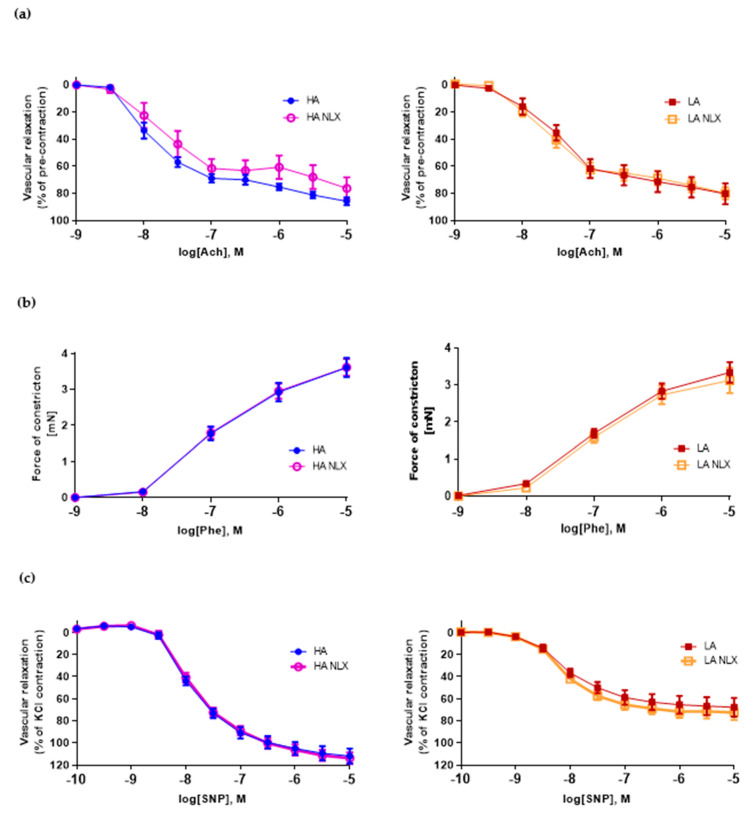
(**a**) Vascular relaxation of aortas from HA and LA mice and aortas treated in vitro with naloxone in response to acetylcholine (*n* = 4–10); (**b**) vascular constriction in aortas from HA and LA mice and aortas treated in vitro treated with naloxone in response to increasing doses of phenylephrine (*n* = 4–10); (**c**) endothelium-independent relaxation of aortas from HA and LA mice and aortas treated in vitro with naloxone in response to increasing doses of SNP (*n* = 4–10).

To investigate role of opioid receptors located in vasculature on the function of the aorta, we performed an in vitro treatment of aortic segments with NLX. Naloxone treatment in both lines did not modify the vascular relaxation induced by acetylcholine (Figure 5a). Neither vascular constriction dependent on phenylephrine was affected (Figure 5b). Additionally, the response to SNP was not statistically different after NLX pre-treatment in both lines (Figure 5c). Therefore, we performed an analysis of opioid receptors in the aorta of those mice. The expression of kappa, delta and mu opioid receptors mRNA was undetectable by RT-qPCR in the aorta from both HA and LA mice (data not shown).

## 3. Discussion

Despite extensive studies in the field of cardiovascular effects related to opioid intake and exogenous opioids [20,21,22], little is known about role of endogenous opioids on vasculature function and BP level. In the study presented herein, we take advantage of a unique model of mice with high and low activity of the endogenous opioid system to present the role of endogenous opioids on the cardiovascular system. Mice selected for high (HA) and low (LA) swim stress-induced analgesia (SSIA) show substantial differences in the magnitude of the antinociceptive response to stress and when treated with exogenous opioids. HA mice express higher peripheral β– endorphin levels, whereas LA mice have low sensitivity to morphine. However, no striking differences in opioid receptor expression or binding were noted between HA or LA mice [16,23].

In our study, mice with low activity of the endogenous opioid system had significantly increased systolic BP in comparison to mice with high activity of the opioid system; however, comparing those data to human guidelines, those mice would be considered as prehypertensive [24]. Both endogenous and exogenous opioids can regulate vascular functions. The agonism of opioid receptors by biphalin decreased BP levels in SHRs [14]. In another study, loperamide, a peripheral opioid receptor agonist, lowered the mean arterial pressure in the same model via the μ(2)-opioid receptor-dependent cAMP-PKA pathway. Moreover, loperamide treatment induced vascular relaxation by opening K(ATP) channels [25]. Interestingly, the hypotensive effect of endogenous opioids which we have observed was strictly related to opioid receptors. The infusion of NLX to HA mice led to hypertension development. Interestingly, in LA mice, NLX treatment did not induce significant changes in BP levels. These data are in line with observations made on human subjects, where NLX increased BP responses during psychological stress in young adults with a low causal BP, but had no pressor effect in subjects with a high casual BP [26]. These results suggest that the opioidergic inhibition of sympathetic nervous system responses may be deficient in patients at risk for essential hypertension. Additionally, the model-dependent effect of NLX was described in three different rat hypertension models. The stimulation of opioid receptors by biphalin decreased the BP level in SHR rats but not in the two other rat hypertension models (uninephrectomized rats on a high-salt diet or angiotensin II-induced hypertension). Biphalin also did not change the BP level in normotensive controls, WKY and Sprague Dawley rats [15]. Considering this, the mouse model we have used in our study has some advantages compared to the models described above. Firstly, in the HA and LA model there is no induction of hypertension by pharmacological or surgical modifications. Moreover, mice selected for high activity of the opioid endogenous system exert some neurological symptoms which are also present in hypertensive patients and which are absent in other models. For example, NLX selectively induces high levels of anxiety- and depressive-like behaviors in HA mice [16]. It is known that patients with depression and/or anxiety represent a particularly vulnerable population as they are at higher risk for developing hypertension [27]. Moreover, hypertension increases memory impairment risk [28], which was also observed in HA mice treated with NLX [29]. Additionally, clinical trials with NLX describe that the antagonism of opioid receptors leads to an increase in BP level, which can be used for shock treatment [30]. These data indicate that NLX’s effect on BP level we have observed in HA mice is consistent with mentioned data from human studies [26,30,31]. However, mechanisms which regulate BP level by NLX are not fully understood yet.

Interestingly, despite the elevated BP level in LA mice, we have not observed vascular dysfunction measured as a response to acetylcholine at the prehypertensive state. However, the observed shift of curve to the right in LA mice may suggest vascular dysfunction initiation. It is known that both small and large artery disease might precede the development of hypertension [32].

To identify the role of opioid receptors located in vasculature on vascular function, we performed an analysis of aortic segments’ function, treated in vitro with NLX. We did not observe changes in vascular contraction in response to phenylephrine and vascular relaxation in response to acetylcholine, even though SNP treatment did not exert any changes in vascular response after the NLX treatment of aortic segments from LA and HA mice. Therefore, we performed an analysis of opioid receptors in the aorta of those mice. The expression of kappa, delta and mu opioid receptors mRNA was undetectable by RT-qPCR in aortas from both HA and LA mice (data not shown). In contrast, some studies describe the expression of opioid receptors on endothelium in the vasculature of rats and humans [33,34]. This finding may explain that the observed effect of BP increase in HA mice after NLX treatment is mediated by an opioid receptor blockade not related to vasculature. It may suggest that NLX’s effect on BP level can be explained only by binding to opioid receptors in the central nervous system, which needs to be confirmed in further studies using naloxone methiodide, which does not cross the blood–brain barrier.

NO plays a fundamental role in the regulation of vascular relaxation. NO is a potent vasodilator produced by the endothelium under basal conditions and in response to a variety of agonists. It diffuses from the endothelium to the underlying vascular smooth muscle, where it causes relaxation through the activation of soluble guanylyl cyclase, causing an increase in 3,5-cyclic guanosine monophosphate (cGMP) [12]. In the aorta, NO is produced mainly by endothelial nitric oxide synthase (eNOS) from L-arginine. Interestingly, LA mice express more eNOS in the aorta than HA mice. However, a limiting factor in NO-dependent relaxation is guanylyl cyclase. In our study, we observed a decreased level of Gucy1A3 in LA mice in comparison to HA mice. Therefore, in spite of a higher expression of eNOS, LA mice have elevated BP. However, studies on endothelial cells and animal models of hypertension have found that eNOS expression elevates in vasculature in response to mechanical forces such as shear stress or BP [35,36,37]. It might be a compensatory mechanism leading to the elevation of NO and relaxation.

Not only nitric oxide synthesis is affected in LA mice but also signaling in the downstream of NO. We also have observed that vascular impairment independent of the endothelium appears in the aorta of LA mice. A similar effect was observed in an angiotensin II-induced model of hypertension [38]. We believe that this effect is mostly related to guanylyl cyclase function. The expression of *Gucy1a3* mRNA in the aorta of LA mice was lower compared to HA. We observed that the regulation of *Gucy1a3* expression is possible through opioid receptor signaling. In HA mice, NLX treatment significantly diminished the expression of *Gucy1a3* in the aorta. It is already known from the literature that soluble guanylyl cyclase may play a role in antinociception by regulating cGMP production [39,40]. It has been described that all opioid receptor agonists (µ, δ and κ) may be involved in NO/cGMP-dependent antinociception and the effect was reversed after guanylyl cyclase inhibitor administration [41,42,43]. However, in our model, higher levels of stress induced analgesia in HA mice were not related to NO. Additionally, sildenafil, which is inhibitor of 5-phosphodiesterase which hydrolyses active cGMP to non-active GMP, was found to play role in antinociception. Sildenafil itself induces antinociception and in combination with morphine enhances its antinociceptive effect [44]. Interestingly, differences in the analgesia level in HA and LA mice are not related to NO. L-NAME treatment (in different doses) of LA and HA mice had no effect on the pain threshold level measured by the TF test and HP test (data not published). One group has described that guanylyl cyclase expression in cerebral regions may be regulated by µ opioid receptor stimulation in rats physiologically dependent on morphine [45]. However, nobody before has described effect of opioid activity on guanylyl cyclase expression in the aorta and referred these data to BP level and vascular function. Further studies are needed to explain the mechanism of guanylyl cyclase expression regulation in the aorta dependent of opioid receptor stimulation.

Summarizing the endogenous opioid system may play a beneficial role in BP regulation. High activity of the endogenous opioid system may regulate guanylyl cyclase expression, leading to the better relaxation of vessels. Guanylyl expression may be modified by opioid receptor antagonists such as NLX. Moreover, we have found that this effect is not mediated by opioid receptors in the aorta. We believe that the observed effect is mediated via the activation of the opioid system in the central nervous system, but it needs further studies in this topic.

## 4. Materials and Methods

### 4.1. Selection Protocol for the HA and LA Lines

Outbred Swiss-Webster mice of either sex, 2 min after completion of 3-min swimming in 20 °C water, were screened for the latency of a nociceptive reflex on a HP test at 56 °C. Those displaying the longest (50–60 s) and the shortest (<10 s) post-swim latencies of the hind paw flick or lick response (whichever occurred first) were selected as progenitors of the HA and the LA lines [17]. A similar procedure was repeated in each offspring generation, but only subjects displaying the longest and the shortest post-swim hot plate latencies were mated to maintain the lines. Experiments on live mice were conducted according to the 2010/63/UE directive and upon ethical clearance (decision no. WAW2/180/2019 and WAW2/058/2020) received from the II Local Ethics Committee for Animal Experimentation in Warsaw.

### 4.2. Measurement of Blood Pressure

Blood pressure was measured before and during the infusion of naloxone using a non-invasive tail cuff measurement system (BP-2000—Blood Pressure Analysis System, Visitech Systems, Apex, NC, USA). Before minipump implantations, mice were trained to tail cuff blood pressure measurements for one week. After minipump implantations, BP was recorded for another 7 days. One session of BP measurement included 5 preliminary measurements and 10 actual measurements which were analyzed. Preliminary measurements were performed in order to allow the animals to warm up sufficiently to produce a good blood flow in the tail. We have not observed specific behavior in HA mice after naloxone treatment.

### 4.3. Hot-Plate Test

During the HP test, individuals were placed in a transparent plexiglass cylinder (15 cm in diameter) situated on a metal square plate heated to 56 °C. Latency was assessed by a blindfolded experimenter with use of stopwatch. Only strong reactions of paw flitching, licking or jumping were considered as nocifensive responses. To avoid burns of mice, the cut-off time was set to 60 s. Measurements were taken before treatment (0 min) and 5, 15, 30, 60 and 120 min after swimming trial.

### 4.4. Tail-Flick Test

The tail-flick (7360 Tail-flick Unit (Ugo Basile, Gemonio, Italy)) test was performed in the same timepoints as hot-plate test. Briefly, animals were restrained with a cotton cloth. The tail was placed at 2/3 of its length over a radiant heat source. Withdrawal reaction latency was measured by built-in timer. Each measurement was taken three times. The cut-off time was set to 10 s to avoid severe burns. Measurements were taken before treatment (0 min) and 5, 15, 30, 60 and 120 min after swimming trial.

### 4.5. Naloxone Administration

Naloxone was administered to HA and LA mice to obtain the continuous blockade of opioid receptors. Mice were treated with naloxone hydrochloride (Sigma-Aldrich, St. Louis, MO, USA) administrated by minipump (1 mg/kg/h) for 7 days with continuous BP monitoring.

### 4.6. Determination of Vascular Function

Thoracic aortas after isolation were cut into 3 mm ring segments and mounted on isometric wire myographs (Danish Myo Technology, Aarhus, Denmark) in physiological saline solution and continuously gassed with a mixture of 95% O_2_ and 5% CO_2_ at 37 °C. Following 60 min of equilibration, the contractility of arterial segments was assessed by the addition of KCl solution (120 mM). The relaxation of the vessels was induced by acetylcholine in arteries pre-contracted with phenylephrine as we described before [20,21]. For the contraction curve, phenylephrine was used in rising concentrations from 10^−9^ to 10^−5^ M. To investigate vascular relaxation independent of the endothelium, SNP was used after NOS inhibition with L-NAME. Additional segments of aortas were incubated with NLX (3 × 10^−5^ M) following each curve.

### 4.7. Measurement of mRNA Expression

RNA from aortas was obtained using RNeasy Lipid Tissue Mini Kit (Qiagen, Hilden, Germany). Total RNA was measured by Nanodrop 2000 (Thermo Fisher Scientific, Waltham, MA, USA). Reverse transcription of 1 μg of RNA was performed using the High Capacity cDNA Reverse Transcription Kit (Applied Biosystems, Foster City, CA, USA). The expression of *Gucy1a3*, *Oprk1*, *Oprm1*, *Oprd1* at mRNA level in the aorta was analyzed using TaqMan^®^ probes (Thermo Fisher Scientific) and the TaqMan^®^ Real-Time PCR Master Mix (Thermo Fisher Scientific). Reactions were prepared and run on 96-well plates on the Applied Biosystems^®^ 7500 Real-Time PCR according to standard protocol. Calculations were made using SDS Software 2.4. Data were normalized to levels of *Tbp* mRNA and relative quantification was calculated.

### 4.8. Western Blot

Extracts of the aortas were prepared for protein analysis. Tissues were suspended in T-PER™ Tissue Protein Extraction Reagent buffer (Thermo Fisher Scientific, Waltham, MA, USA) with the addition of a protease inhibitor cocktail (Sigma-Aldrich, St. Louis, MO, USA) were homogenized with FastPrep-24 (MP Biomedicals, Irvine, CA, USA). After centrifugation, supernatants were collected and the protein concentration was determined by the BCA assay (Thermo Fisher Scientific, Waltham, MA, USA). Protein extracts were subjected to SDS-PAGE electrophoresis. The transfer was performed using PVDF membranes previously activated in methanol. The membranes were blocked with 5% skim milk. After overnight incubation with the primary antibody 1:1000 (Actin Monoclonal Antibody, Thermo Fisher Scientific, Waltham, MA, USA and GUCY1A3 Polyclonal antibody, Proteintech, Chicago, IL, USA) at 4 °C, membranes were washed and incubated with horseradish peroxidase-conjugated secondary antibodies (goat, anti-mouse IgG and goat anti-rabbit IgG, Sigma-Aldrich, St. Louis, MO, USA), 1:10,000, at room temperature for 1 h and then visualized using SuperSignal chemiluminescent substrate (Thermo Fisher Scientific, Waltham, MA, USA). Western blots were acquired using the ChemiDoc XRS+ (Bio-Rad). Analyses were made using Image Lab (Bio-Rad) software. All targets were normalized first to the loading control (β-actin).

### 4.9. Data and Statistical Analysis

For the comparison of three or more independent groups, one-way ANOVA was used with a Student–Newman–Keuls post hoc test. For comparison of two groups, unpaired two-tailed *t*-tests were used. For comparisons of vascular function in organ chamber experiments, repeated measures ANOVA with post hoc Bonferroni correction was used. *p* values < 0.05 were considered significant. GraphPad Prism and PowerPoint were used for data visualization.

## Data Availability

The data presented in this study are available on request from the corresponding author.
